# Formes évolutives postérieures de la persistance de la vascularisation fœtale bilatérale à l'hôpital régional de Gao

**DOI:** 10.11604/pamj.2018.31.215.16513

**Published:** 2018-12-03

**Authors:** Ibrahima Conaré, Mahamadou Mallé

**Affiliations:** 1 Hôpital Hangadoumbo Moulaye Touré de Gao, Gao, Mali

**Keywords:** Persistance de la vascularisation fœtale, exotropie, complications évolutives, Persistence of fetal vasculature, exotropia, evolving complications

## Abstract

The persistence of fetal vasculature (PFV) is an abnormal resorption in hyaloid vascular system. We report the case of a 2-year old child with evolutionary posterior form of bilateral vasculature presenting with leukocoria in the right eye. Clinical examination showed bilateral leukocoria, exotropia in the right eye and an exotropia in the left eye. Fundus examination of the right eye showed fibrovascular retinal band stretched to the ora serrata associated with total retinal detachment and peripheral pigment changes. Fundus examination of the left eye showed fibrovascular retinal band stretched from the papilla to the ora serrata, associated with partial inferior, anterior temporal detachment and peripheral pigment changes

## Image en médecine

La persistance de la vascularisation fœtale (PVF) est une anomalie de la résorption du système vasculaire hyaloïdien. Formes évolutives postérieures bilatérales de la persistance chez un garçon âgé de 2 ans qui a consulté pour une leucocorie de l'œil droit. L'examen a retrouvé une leucocorie bilatérale, une esotropie de l'œil droit et une exotropie de l'œil gauche. Le fond d'œil droit retrouve une bande fibrovasculaire rétinien tendu jusqu’à l’ora serrata, associé à un décollement total de la rétine et des remaniements pigmentaires périphériques. Le fond d'œil gauche note une bande fibrovasculaire rétinien tendu de la papille à l’ora serrata, associée à un décollement partiel temporal inférieur en antérieur et des remaniements pigmentaires périphériques.

**Figure 1:**
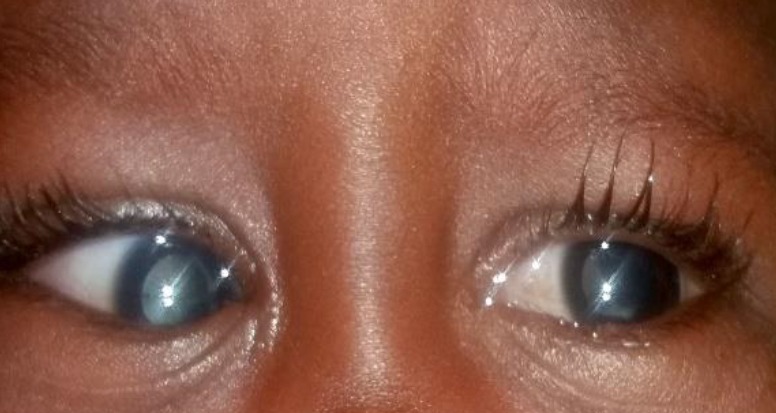
formes évolutives postérieures de la persistance de la vascularisation

